# Supplementary material for: Process evaluation of the BASIL+ trial: A behavioural activation approach to addressing low mood and depression among older people with long-term conditions
Source: PLOS Ment Health. 2025 Jan 23;2(1):e0000105. doi: 10.1371/journal.pmen.0000105 (PMC12798173; doi:10.1371/journal.pmen.0000105)
Supplement: Attachment — Submitted filename: PMEN-D-24-00283 Response to Reviewers FINAL.docx [file pmen.0000105.s001.docx]

**PMEN-D-24-00283**

**Response to Reviewers Comments**

| **Editor/Reviewers Comment** | **Authors Response** |
| --- | --- |
| **Editor** |  |
| The intervention is a complex intervention with at least three active ingredients / mechanisms. Refer to the framework for complex intervention evaluation more explictly throughout. <https://www.bmj.com/content/374/bmj.n2061> in particular complex interventions as events in systems. Make more of the interaction between the intervention and its delivery context. e.g. does your evaluation have any implications for theory modification?  L130 Is purposive the right word?  L140 Completers vs non-completers should be a bullet here (and an entry in Table 1) with numbers  L195 Brief explain what deductive and induction means to readers unfamiliar with qualitative research  L230 Reference needed for existing quantitative research on pre-pandemic social isolation (e.g. introduction here <https://www.frontiersin.org/journals/psychology/articles/10.3389/fpsyg.2020.02201/full>)  L232 Please avoid 'speak to' which sounds fluffy and informal L240 Should be non-completers not none-completers L541 Please add references to quantitative findings, even if in press / recently accepted L600 Not clear when you say BASIL+ qualitative study if you mean this paper or another qualitative paper? | We have acknowledged that it is a complex intervention and referred to the framework you provided a link for.  We do not consider our evaluation has implications for theory modification other than highlighting the importance of a flexible, person-centred approach to intervention delivery.  Yes, it is the correct word.  We have changed the wording to make it clear that the purposive sample was of those participants who completed the intervention.  We have revised the wording to assist readers unfamiliar with qualitative research.  Thank you for this suggestion. We have revised the wording to clarify the point. However, we do not feel it would be appropriate to include a reference as we are referring to our findings rather than the wider literature.  We agree – wording now changed  Thank you – now corrected  Now added  We are referring to this paper. We have adjusted the wording to make this clear |
| **Reviewer 1** |  |
| I have very few comments on this paper. It is a rigorous evaluation of the process of the BASIL+ trial. However: I would have liked something more about the 'adaptation' of the (L67) for the setting of COVID in this paper to frame the findings, and comment on whether any further adaptation was found to be necessary, or not. (L102) some explanation of why the collaboration care intervention was considered to be 'light touch' would be helpful to readers unfamiliar with collaborative care. (L229) the section on context of the findings is interesting, but some data to support the statement in the line sentence beginning 'Analysis suggested...' would be helpful. (L337) were there any examples of the reality of lockdown impeding chosen social activities or how these were modified to suit the circumstances given that the potential need to do this was recognised in the protocol? (L507) missing word Finally were there any particular issues that arose in supervision of the BSWs given the potential difficulties arising in such a study during COVID? Supervisors were not interviewed and may have provided some useful insights. | Thank you.  We have added an explanation of the adaptation, which was essentially moving to remote support. Once the trial began not further adaptations were made.  We have added further explanation.  Thank you. We have changed the wording to make it clear that this statement relates to our findings.  At the time of the study there were restrictions on social mixing but not full ‘lockdown’. I don’t think we can say more here as participants simply talked about what they were able to do.  The training and support for BSW will be the subject or another paper so we have not discussed it here in any depth. Supervision of the BSW was by telephone and did not change. We did not interview the supervisors as they were members of the BASIL+ study team and so it would not have felt appropriate. |
| **Reviewer 2** |  |
| Overall, this is a very clear and well written paper reporting a qualitative process evaluation. The introduction describes the background well, the methods provide a detailed description of the study process. The themes developed in the results section are interesting, succinct and clearly described. The discussion is appropriate and offers further contextualisation of the insights offered in the results. Consequently, I have little to suggest by way of improvements.  One area to consider perhaps is that, as written, it is a very positive report of the intervention, its use and its impact. Which left me wondering if there was more to be said regarding any participants who did not get on well with the intervention? We are told that in most cases the intervention had a positive impact. What happened where it did not? Were these the participant who had taken part primarily for altruistic reasons rather than low mood (we also find this in our research), the non-completers? Were there any completers who didn’t like it? I understand if not, but it would be useful just to cover this a little more.  One other minor aspect to expand on is in the analysis section. It says a deductive and inductive approach was adopted. Could the authors expand? In what way was the approach to analysis deductive, that wasn’t clear to me.  As it stands, this process analysis represents an important and useful contribution to this area, complementing the trial publications. Expanding on the above issues may further strengthen the paper. | Thank you for your positive comments.  The majority of participants who completed the intervention got something positive from it. We wanted to include more non-completers, to understand why people dropped out and the things they didn’t like. However, this proved difficult as non-completers often chose to withdraw from the whole trial and so we could not contact them for the process evaluation. We have tried to cover the views of those we could interview on page 13 but we agree the data is limited.  We agree. We expanded this section to explain more clearly how our analysis was both deductive and inductive.  Thank you. |
